# Supplementary material for: Perinatal granulopoiesis and risk of pediatric asthma
Source: eLife. 2021 Feb 10;10:e63745. doi: 10.7554/eLife.63745 (PMC7889076; doi:10.7554/eLife.63745)
Supplement: Transparent reporting form [file elife-63745-transrepform.pdf]

1st Floor
24 Hills Road
Cambridge CB2 1JP, UK

P 01223 855340
W elifesciences.org
T @elife

eLife's transparent reporting form

We encourage authors to provide detailed information *within their submission* to facilitate the interpretation and replication of experiments. Authors can upload supporting documentation to indicate the use of appropriate reporting guidelines for health-related research (see EQUATOR Network), life science research (see the BioSharing Information Resource), or the ARRIVE guidelines for reporting work involving animal research. Where applicable, authors should refer to any relevant reporting standards documents in this form.

If you have any questions, please consult our Journal Policies and/or contact us: editorial@elifesciences.org.

Sample-size estimation

- You should state whether an appropriate sample size was computed when the study was being designed
- You should state the statistical method of sample size computation and any required assumptions
- If no explicit power analysis was used, you should describe how you decided what sample (replicate) size (number) to use

Please outline where this information can be found within the submission (e.g., sections or figure legends), or explain why this information doesn't apply to your submission:

No sample sizes were predetermined. Samples were accrued based on availability from existing cohorts. Limitations regarding power and the current study design are discussed on line 361.

Replicates

- You should report how often each experiment was performed
- You should include a definition of biological versus technical replication
- The data obtained should be provided and sufficient information should be provided to indicate the number of independent biological and/or technical replicates
- If you encountered any outliers, you should describe how these were handled
- Criteria for exclusion/inclusion of data should be clearly stated
- High-throughput sequence data should be uploaded before submission, with a private link for reviewers provided (these are available from both GEO and ArrayExpress)

Please outline where this information can be found within the submission (e.g., sections or figure legends), or explain why this information doesn't apply to your submission:

Findings of the meta-analysis were replicated in an independent prospective cohort, UIH cohort. mRNA findings were assessed for correspondence at protein and cellular level in an independent cohort, Olin et al. Protein findings were further replicated in Project Viva. Datasets for the meta-analysis were excluded on the basis of homogeneous meta-data to which not testing can be applied. No data was excluded from replication analyses (Olin et al, UIH, Project Viva).

eLife Sciences Publications, Ltd is a limited liability non-profit non-stock corporation incorporated in the State of Delaware, USA, with company number 5030732, and is registered in the UK with company number FC030576 and branch number BR015634 at the address 1st Floor, 24 Hills Road, Cambridge CB2 1JP | August 2014

Statistical reporting

- Statistical analysis methods should be described and justified
- Raw data should be presented in figures whenever informative to do so (typically when N per group is less than 10)
- For each experiment, you should identify the statistical tests used, exact values of N, definitions of center, methods of multiple test correction, and dispersion and precision measures (e.g., mean, median, SD, SEM, confidence intervals; and, for the major substantive results, a measure of effect size (e.g., Pearson's r, Cohen's d)
- Report exact p-values wherever possible alongside the summary statistics and 95% confidence intervals. These should be reported for all key questions and not only when the p-value is less than 0.05.

Please outline where this information can be found within the submission (e.g., sections or figure legends), or explain why this information doesn't apply to your submission:

All statistical reporting can be found in the results section. Methods regarding statistical tests used can be found in the four sections under statistical analysis in the methods section.

(For large datasets, or papers with a very large number of statistical tests, you may upload a single table file with tests, Ns, etc., with reference to sections in the manuscript.)

Group allocation

- Indicate how samples were allocated into experimental groups (in the case of clinical studies, please specify allocation to treatment method); if randomization was used, please also state if restricted randomization was applied
- Indicate if masking was used during group allocation, data collection and/or data analysis

Please outline where this information can be found within the submission (e.g., sections or figure legends), or explain why this information doesn't apply to your submission:

No randomization. Co-variates were adjusted for when assessing odds of asthma and association with FEV1/FVC. Subjects with missing data regarding outcomes were used to assess for sampling bias, see Figure S5 and supplemental data 7.

Population Characteristics are described in supplemental data 5 for the UIH cohort and in supplemental data 7 for Project Viva.

Line 428: Women seeking prenatal care at the University of Illinois at Chicago (UIC) Center for Women's Health were recruited as volunteers in their third trimester (29–33 weeks of gestation) between 2014 and 2017. The cohort profile has been previously described by Koenig and colleagues (59). Inclusion criteria as follows: singleton pregnancy; naturally conceived pregnancy; 17–45 y of age; pre-pregnancy BMI ≥18.5; < 34 weeks of gestation; sufficient fluency in English to provide consent and complete the study; and ability to independently provide consent. Exclusion criteria as follows: live birth or another pregnancy (including ectopic and molar pregnancies) in the previous 12 months; preeclampsia; gestational diabetes mellitus or previously diagnosed type 1 or type 2 diabetes; autoimmune disorder; current or previous premature rupture of membranes or chorioamnionitis; previous spontaneous premature birth; current bacterial or viral infection; current steroid or anti-inflammatory treatment; history of bariatric surgery; malabsorptive condition (e.g., celiac disease); current hyperemesis; hematologic disorder (e.g., sickle cell anemia or trait, hemochromatosis); current tobacco use; alcohol consumption or illicit drug use; and current use of medications that decrease nutrient absorption (e.g., proton pump inhibitors).

Line 401: Volunteers were recruited from women attending their first prenatal visit at one of 8 practices of Atrius Harvard Vanguard Medical Associates. The exclusion criteria were multiple gestation, inability to answer questions in English, gestational age ≥ 22 weeks at recruitment, and plans to move away before delivery. The cohort profile was previously described by Oken and colleagues (57).

Additional data files ("source data")

- We encourage you to upload relevant additional data files, such as numerical data that are represented as a graph in a figure, or as a summary table
- Where provided, these should be in the most useful format, and they can be uploaded as "Source data" files linked to a main figure or table
- Include model definition files including the full list of parameters used
- Include code used for data analysis (e.g., R, MatLab)
- Avoid stating that data files are "available upon request"

Please indicate the figures or tables for which source data files have been provided:

Figure 2A-C source data is located in Supplemental Tables 1-4.
